# Approach to growth hormone therapy in children with chronic kidney disease varies across North America: the Midwest Pediatric Nephrology Consortium report

**DOI:** 10.1186/s12882-017-0599-1

**Published:** 2017-05-30

**Authors:** Oleh M. Akchurin, Amy J. Kogon, Juhi Kumar, Christine B. Sethna, Hoda T. Hammad, Paul J. Christos, John D. Mahan, Larry A. Greenbaum, Robert Woroniecki

**Affiliations:** 1 Weill Cornell Medicine, New York, USA; 2 Ohio State University / Nationwide Children’s Hospital, Columbus, USA; 3 Hofstra Northwell School of Medicine / Cohen Children’s Medical Center of New York, Hempstead, USA; 4 Emory University / Children’s Healthcare of Atlanta, Atlanta, USA; 5 Stony Brook University, Stony Brook, USA

**Keywords:** Short stature, Linear growth, Chronic kidney disease, Growth hormone, Standards of care, Survey

## Abstract

**Background:**

Growth impairment remains common in children with chronic kidney disease (CKD). Available literature indicates low level of recombinant human growth hormone (rhGH) utilization in short children with CKD. Despite efforts at consensus guidelines, lack of high-level evidence continues to complicate rhGH therapy decision-making and the level of practice variability in rhGH treatment by pediatric nephrologists is unknown.

**Methods:**

Cross-sectional online survey electronically distributed to pediatric nephrologists through the Midwest Pediatric Nephrology Consortium and American Society of Pediatric Nephrology.

**Results:**

Seventy three pediatric nephrologists completed the survey. While the majority (52.1%) rarely involve endocrinology in rhGH management, 26.8% reported that endocrinology managed most aspects of rhGH treatment in their centers. The majority of centers (68.5%) have a dedicated renal dietitian, but 20.6% reported the nephrologist as the primary source of nutritional support for children with CKD. Children with growth failure did not receive rhGH most commonly because of family refusal. Differences in initial work-up for rhGH therapy include variable use of bone age (95%), thyroid function (58%), insulin-like growth factor-1 (40%), hip/knee X-ray (36%), and ophthalmologic evaluation (7%). Most pediatric nephrologists (95%) believe that rhGH treatment improves quality of life, but only 24% believe that it improves physical function; 44% indicated that rhGH improves lean body mass.

**Conclusions:**

There is substantial variation in pediatric nephrology practice in addressing short stature and rhGH utilization in children with CKD. Hence, there may be opportunities to standardize care to study and improve growth outcomes in short children with CKD.

**Electronic supplementary material:**

The online version of this article (doi:10.1186/s12882-017-0599-1) contains supplementary material, which is available to authorized users.

## Background

Growth impairment remains common in pediatric chronic kidney disease (CKD), despite advances in care of children with CKD [1]. In the North American Pediatric Renal Trials and Collaborative Studies (NAPRTCS) CKD registry, 36.9% had height standard deviation scores (SDS) < −1.88 at the time of enrollment [2]. In a more recent study cohort of North American children with mild to moderate CKD, the median height SDS was −0.55 at study entry [3]. In a European cohort of patients who received renal replacement therapy during childhood, the median final adult height SDS was −1.65 [4].

Short stature is associated with a lower quality of life (QOL) in children with CKD [5], and this lower QOL persists into adulthood [6]. Treatment with recombinant human growth hormone (rhGH) is associated with improved physical and social functioning according to parental reports [7]. It would be reasonable to expect that rhGH treatment improves QOL in children with CKD, but no direct evidence is currently available to support this assumption. In addition, rhGH therapy may provide health benefits not directly related to improved linear growth, such as increased lean body mass, improved appetite and nutrition, increased physical function and decreased fatigue [8, 9]. It is unknown if pediatric nephrologists consider these potential benefits of rhGH therapy in their therapeutic decisions.

Treatment with rhGH is FDA-approved in short children with CKD in the U.S. and rhGH has been used in children with CKD for over 25 years [10]. Yet, rhGH utilization in short children with CKD is quite low in both North America and many European countries [11, 12]. Some of the reported obstacles for use of rhGH are family refusal, non-compliance, severe hyperparathyroidism, poor nutrition, younger age, neurologic impairment, malignancy or scheduled transplant [13]. However, the perspectives of pediatric nephrologists about the barriers to rhGH utilization have never been systematically investigated.

There is a paucity of high-level evidence to direct evaluation of short children with CKD prior to prescription of rhGH therapy. Requirements for diagnostic testing related to rhGH insurance approval vary, with some insurers in the U.S. only requiring a bone age for short children over 12 years old whose height is below the 3rd percentile and an estimated GFR below 75 ml/min/1.73m^2^. The U.S. physicians have considerable discretion in determining testing prior to seeking rhGH insurance approval. The most recent consensus paper written by a group of U.S. experts is now over 10 years old [9] and suggests that optimal preparation for starting rhGH therapy requires efforts at addressing modifiable factors related to growth failure and baseline hip and knee X-rays, funduscopic evaluation, and thyroid studies. There is no consensus on the value of baseline and subsequent GH-insulin like growth factor-1 (IGF-1) axis assessments in monitoring rhGH therapy in children with CKD.

RhGH treatment, in part due to its high cost, is further complicated by the logistic challenges involved in its prescription. Insurance approval, nutritional evaluation and monitoring, management of other CKD complications (such as CKD mineral bone disorder, anemia, and acidosis), optimization of dialysis, and potential involvement of endocrinology, all require system based approaches to rhGH therapy in order to deliver well organized care within the unique environment and available resources of each individual institution. These challenges and varying resources may contribute to practice variability in the prescription of rhGH.

We hypothesized that there is substantial variability in practice patterns and resources available to support rhGH treatment among pediatric nephrology centers, which may affect treatment decisions. Hence, we analyzed the key characteristics of rhGH treatment approaches by surveying pediatric nephrologists in the US and Canada.

## Methods

Survey items were developed and piloted by the Midwest Pediatric Nephrology Consortium (MWPNC) CKD working group, reviewed and approved by the MWPNC Protocol Review Committee. Institutional Review Board (IRB) approval was obtained at the primary site (Weill Cornell Medicine). The survey (Additional file 1: Item S1) was distributed to physicians by the MWPNC and American Society of Pediatric Nephrology (ASPN) from October 2015 to January 2016 via email. The MWPNC involves 60 pediatric nephrology centers who work together in collaborative pediatric nephrology research. The ASPN is the premier organization of pediatric nephrologists in North America with approximately 150 pediatric nephrology groups/centers, including all of the MWPNC sites except those in Canada. The survey was anonymous and was not offered to fellows. Data were reported as median [interquartile range (IQR)] and frequency (percent). Responses were compared between “small” (4 or fewer nephrologists per center) and “large” (more than four nephrologists) centers by the Wilcoxon rank-sum test or Fisher’s exact test, as appropriate. Centers were categorized based on the median number of nephrologists per center in the cohort. All *p*-values were two-sided with statistical significance evaluated at the 0.05 alpha level. All analyses were performed in SAS Version 9.4 (SAS Institute, Inc., Cary, NC).

## Results

### Study participants

The survey was offered to active members of the MWPNC who previously provided their emails to the MWPNC database (*n* = 200). Individual response rate was 33% and response rate per institution (the percentage of institutions with at least one response) was >50%. The response rate per institution could not be calculated precisely because reporting institution was optional and seven nephrologists elected not to report their institution. In total, seventy-three pediatric nephrologists responded to the survey, representing 26 states and D.C. in the U.S., and two Canadian provinces. Sixty-six participants, who reported their institution, represented 41 pediatric nephrology practices, 23 small (4 or less pediatric nephrologists per practice) and 18 large (5 or more pediatric nephrologists per practice). There were on average 1.6 responses per practice (range 1–5). There were no significant differences in the number of responses per practice between small (1.5 responses) and large (1.7 responses) practices.

### Practice characteristics

The median number of pediatric nephrologists per center was 4 [IQR: 3, 8], and the median number of years spent in practice after fellowship was 9 [IQR: 4, 15] (Table 1). Most nephrologists (60.3%) reported taking care of 1–5 patients receiving rhGH. In small centers, 17.5% of nephrologists did not have any patients treated with rhGH, which was not seen in large centers (Table 1). Institutions represented by nephrologists who had more than 5 rhGH patients also had a higher number of dialysis patients compared to institutions represented by nephrologists who had five or fewer rhGH patients (15 [10, 27] vs. 9 [4, 18], respectively; *p* = 0.03).

**Table 1:** General characteristics of the survey participants by the size of participating centers

	Total (73 responses)	Small centers (40 responses)	Large centers (33 responses)	*p*-value
Number of pediatric nephrologists per center, median [IQR]	4 [3, 8]	3 [3, 4]	8 [7, 10]	
Number of pediatric nephrologists who were in practice for >10 years, n (%)	25 (34)	10 (25)	15 (45)	0.07
Number of patients receiving rhGH, n (%):				0.02^a^
0	7 (9.6)	7 (17.5)	0 (0)	
1–5	44 (60.3)	20 (50.0)	24 (72.73)	
> 5	22 (30.1)	13 (32.5)	9 (27.27)	
Number of patients on dialysis, median [IQR]	12 [5, 18]	5.5 [3, 11]	21.5 [13, 32.5]	<0.001

^a^Fisher’s exact test, IQR-interquartile range [25%, 75%], rhGH- recombinant human growth hormone. All data are shown per center, except the numbers of patients receiving rhGH, which are shown per nephrologist

### Availability of resources to support rhGH treatment program

Resources available for the support of rhGH treatment program were significantly different between small and large centers (Table 2). Thus, while the majority of large centers had a renal dietitian (90.9%), only half of small centers had a renal dietitian (*p* = 0.001). Furthermore, in a third of small centers nutritional needs of children with growth failure and CKD were addressed solely by pediatric nephrologists. Conversely, 43.6% of small centers utilized endocrinology for most aspects of rhGH therapy, compared to 6.3% in large centers (*p* < 0.001). Prior authorization for rhGH therapy was addressed primarily by the nurses (75.3%), with slightly more availability of nursing support for prior authorization in large centers vs. small centers (81.8% vs. 70.0%, respectively, *p* = 0.04) (Table 2).

**Table 2:** Resources available to support growth hormone treatment program by the size of participating centers

	Total (73 responses)	Small centers (40 responses)	Large centers (33 responses)	*p*-value
Nutritional support for short children with pre-dialysis CKD, n (%)				
Renal dietitian Pediatric dietitian Pediatric nephrologist	50 (68.5) 9 (12.3) 15 (20.6)	20 (50.0) 7 (17.5) 13 (32.5)	30 (90.9) 1 (3.0) 2 (6.1)	0.001^a^
Role of endocrinology in rhGH management in CKD, n (%)				
Primary Initial consultation Challenging cases Rarely involved	19 (26.8) 4 (5.6) 11 (15.5) 37 (52.1)	17 (43.6) 3 (7.7) 6 (15.4) 13 (33.3)	2 (6.3) 1 (3.1) 5 (15.6) 24 (75.0)	<0.001^a^
Prior authorization for rhGH, n(%)				
Nurse Attending physician Other	55 (75.3) 8 (11.0) 10 (13.7)	28 (70.0) 3 (7.50) 9 (22.5)	27 (81.8) 5 (15.2) 1 (3.0)	0.04^a^

^a^Fisher’s exact test

### Work up preceding rhGH therapy

**Fig. 1:**
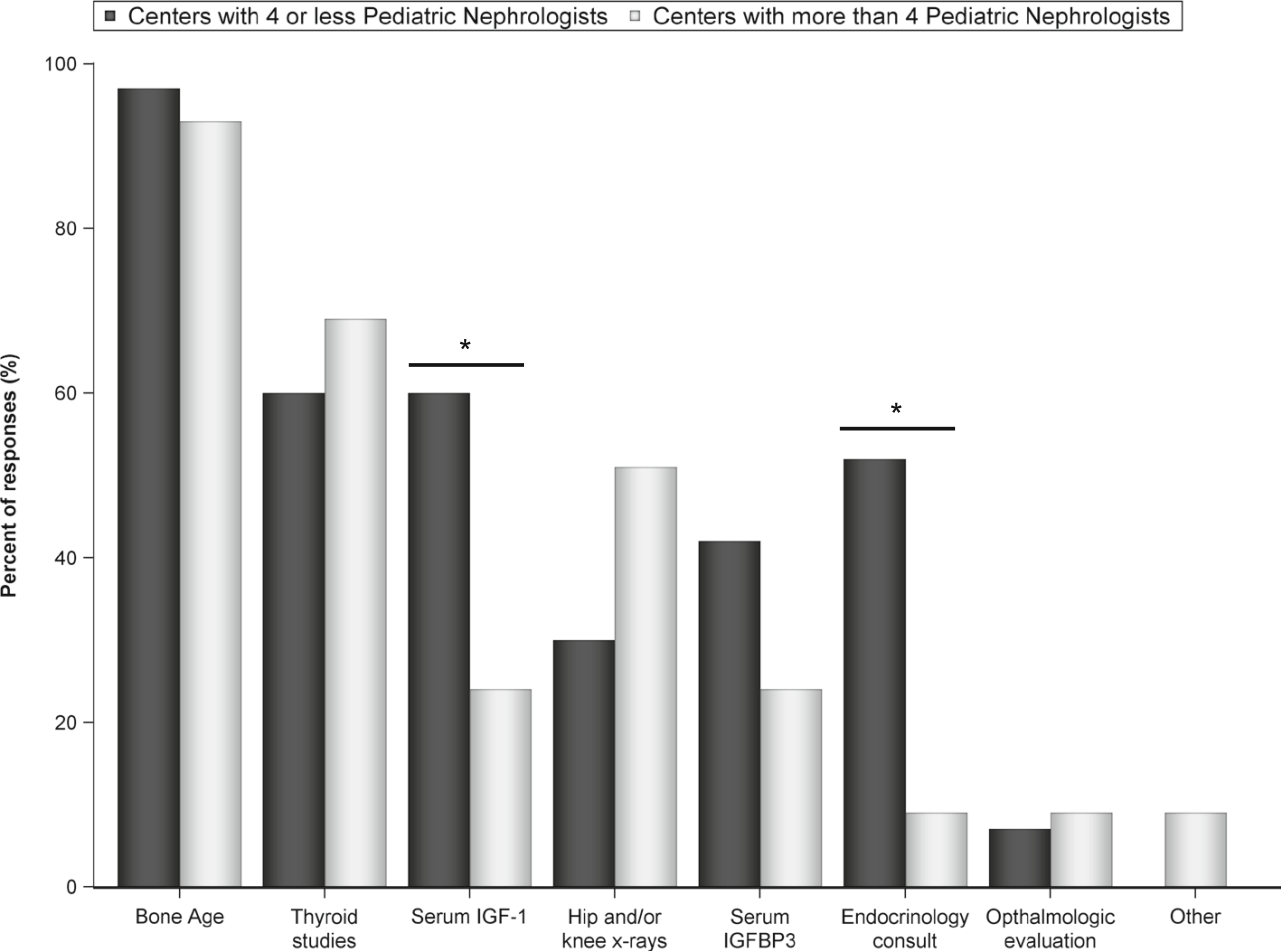
Standard workup preceding growth hormone therapy initiation, reported by study participants (*n* = 73). *p < 0.05

The routine workup for rhGH therapy candidates with CKD differed significantly among centers (Fig. 1). Most pediatric nephrologists routinely obtained bone age, but hip and knee X-rays were obtained by only 39.7%. Approximately half of pediatric nephrologists obtained thyroid studies. Serum IGF-1 was measured by fewer than half of nephrologists, and was ordered more frequently in small centers (*p* = 0.02). About a third of study participants reported ordering IGF-binding protein 3 (IGFBP3), also with a trend toward more frequent use in small centers. Endocrinology consultation was part of the initial workup almost exclusively in small centers (*p* = <0.001). Interestingly, in centers with regular endocrinology involvement, IGF-1 was obtained more frequently (in 64.7% vs. 35.3% in centers with rare endocrinology involvement; *p* = 0.007). Ophthalmologic evaluation was rarely utilized prior to rhGH therapy initiation in both large and small centers (Fig. 1).

### Reported reasons for not prescribing growth hormone to short children with CKD

**Fig. 2:**
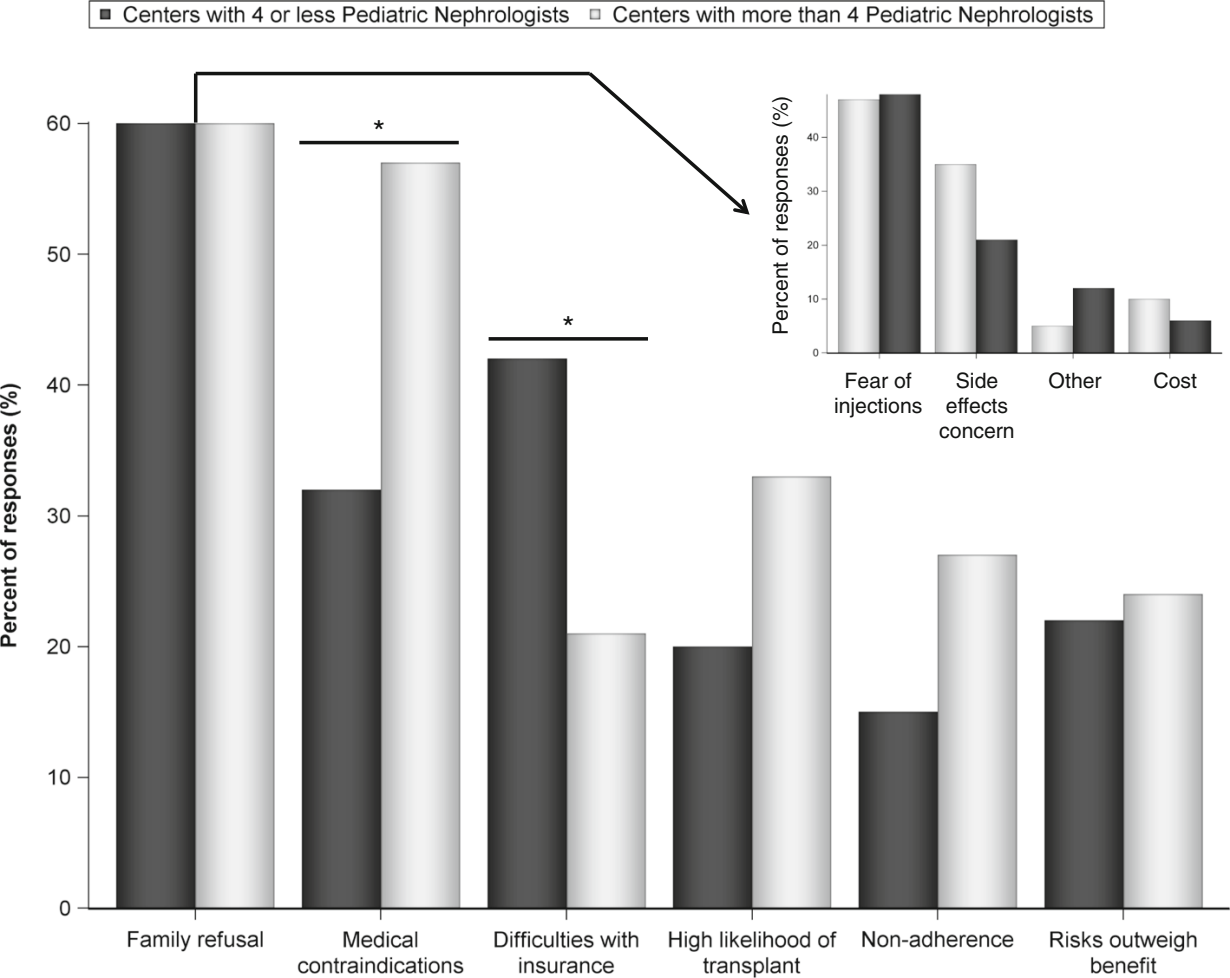
Perceived reasons for short children with chronic kidney disease not receiving growth hormone therapy. The insert shows specific reasons for family refusal. Total number of participants *n* = 73. *p < 0.05

Family refusal was the most common reason for short children not receiving rhGH in both large and small centers (Fig. 2). Fear of injections was the most common reason for family refusal (Fig. 2, insert). Overall, concern about side effects was the second most common reason for family refusal. Medical contraindications for rhGH therapy include active malignancy, uncontrolled severe hyperparathyroidism and closed epiphyseal growth plates, among others [1, 9]. In large centers, medical contraindications were the second most common reason for not prescribing rhGH (*p* = 0.03, difference from small centers). In small centers, the second most common cause was difficulties with insurance approval (*p* = 0.05, difference from large centers).

### Benefits and risks of growth hormone therapy in children with CKD

**Fig. 3:**
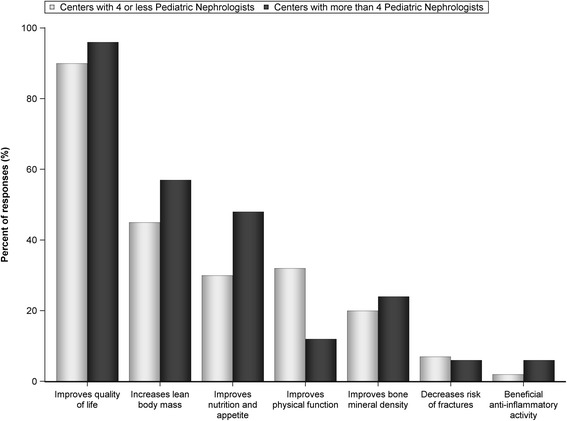
Perceived benefits of growth hormone therapy in children with chronic kidney disease. Total number of participants *n* = 73

The majority of pediatric nephrologists believed that rhGH therapy improves the QOL of short children with CKD (Fig. 3). About 40% of participating nephrologists believed that growth hormone increases lean body mass, and about a third stated that it improves nutrition and appetite. Senior nephrologists were more likely to believe that rhGH improves nutrition and appetite than their junior colleagues (*p* = 0.047). Only 23.3% of nephrologists thought that rhGH improves physical function (Fig. 3). Side effects of rhGH therapy were observed by pediatric nephrologists infrequently (Additional file 2: Figure S1). Headaches and benign intracranial hypertension were reported as the most frequent side effects.

## Discussion

In this study, we describe contemporary pediatric nephrology practice variability in the approaches to growth hormone therapy for short stature in children with CKD. Our analysis was based on a large sample of participating pediatric nephrology centers in North America. Availability and utilization of ancillary resources was a major variable related to the size of the practice.

Available data indicate that rhGH is under-utilized in short children with CKD in the U.S. and Europe. An earlier study conducted by the MWPNC [13] found that 51% of children with CKD whose height fell below the 5th percentile had not received rhGH. In a recent European study including 13 countries, short stature was present in 30.1% of dialysis patients and only 25.1% of these short children were receiving rhGH. Moreover, only 7.6% of the 42.3% of transplant patients with short stature were receiving rhGH [12]. Kidney Disease Outcomes Quality Initiative (KDOQI) guidelines provide limited recommendations for treatment of short stature in pediatric CKD [14]. A group of US experts developed a consensus statement about the assessment and treatment of short stature in pediatric patients with CKD in 2006 [9]. Acceptance of this consensus by the broader pediatric nephrology community and achievement of recommended practices has not been evaluated to date.

The consensus statement suggests evaluation of bone age, hip and knee x-rays, funduscopic exam and thyroid studies prior to rhGH therapy initiation. Bone age, also required by most payers for rhGH approval for children older than 12 year., was a routine part of the workup in most of the centers in this study. Thyroid function tests were reported by less than half of study participants, and hip and knee X-rays by less than third. Ophthalmologic evaluation was very infrequent, despite benign intracranial hypertension being the most frequent observed side effect of rhGH therapy. Serum IGF1 and IGFBP3 were part of the workup in the substantial number of centers, particularly in small centers where endocrinology consultation was more frequent. Overall, our data demonstrate significant variability in approaches to the initial assessment of short children with CKD prior to rhGH treatment initiation, and in many cases substantial differences from the 2006 Consensus Statement.

This study demonstrated significant variability in practice patterns relates to practice group size and resources. The majority of participating pediatric nephrologists cared for 1–5 short children with CKD treated with rhGH, and pediatric endocrinologists managed these patients in almost half of the smaller centers. The opportunities for rhGH management may be less frequent in smaller centers, potentially due to fewer resources resulting in outsourcing to endocrinology. Thus, almost 20% of pediatric nephrologists from small centers reported that they do not have any patients treated with rhGH in their practice. Our results demonstrate various models of interaction between pediatric nephrology and endocrinology in management of short stature in children with CKD. Investigation of whether this variability results in different outcomes was beyond the scope of our study, but would be of interest in future studies.

Both KDOQI and the 2006 Consensus Statement recommend nutritional optimization prior to starting rhGH [9, 14]. Dietary management of children with CKD is complex and time-consuming [15]. Pediatric renal dietitian support seems to be the optimal solution to these challenges. Our data demonstrated that >90% of nephrologists practicing in large centers had renal dietitian support. In contrast, only half nephrologists from small centers had such support. Our study was not designed to confirm whether renal dietitian unavailability led to rhGH underutilization and outcomes differences. However, it suggests that variability in nutritional support should be considered in organizing rhGH treatment programs for short children with CKD and ensuring their success.

In our study, family refusal was the leading cause of short children with CKD not receiving rhGH. This is consistent with an earlier report [13], where family refusal accounted for 18% of short children with CKD not being treated with rhGH. This significant number of refusals may indicate a need for developing specific counseling strategies targeting the issue of refusal. Our data indicated that fear of injections was the number one reason for family refusal, followed by side effect concerns. Alleviating fear of injections seems to be a promising target in increasing use of this therapy. Systematic analysis of treatment satisfaction of children with CKD who completed rhGH therapy may reveal the actual significance of the discomfort from injections. Cost of rhGH treatment was not reported as a significant reason for refusal, but this may be different beyond North America.

Medical contraindications were the second most common reported reason for not receiving rhGH in our study. This reason was reported more frequently by nephrologists practicing in large centers, possibly due to the overall higher complexity of patients with CKD in large centers. Difficulties with insurance approval were the third leading cause for rhGH underutilization. Importantly, insurance problems were reported more frequently in small centers, again suggesting that availability of resources may be affecting care in pediatric nephrology practices. Prior authorization was obtained by the nurses in the majority of both small and large centers but differences in the scope of nephrology nurses’ responsibilities in small vs. large centers may account for some of the insurance difficulties with rhGH approval.

The majority of pediatric nephrologists indicated that rhGH therapy improves QOL of children with CKD. It is important to highlight, however, that we still do not have direct evidence of such a benefit. Short stature in CKD is associated with worse QOL, particularly in the physical functioning domain [5]. Analysis of the large cohort of north American children with CKD showed an association between rhGH use and improved child’s physical and social functioning by parental report [7]. QOL studies in children with non-CKD related short stature treated with rhGH have yielded conflicting results [16, 17]. Evidence of improved QOL with rhGH treatment in CKD is needed, as it may help to impact use of this therapy in short children with CKD. It has been suggested that rhGH therapy may provide additional benefits, such as anabolic effects [8], in children with CKD [9]. More research is needed to investigate these and other potential benefits of rhGH therapy in pediatric CKD population, which in turn may increase the number of short children with CKD taking advantage of rhGH treatment.

Our study has some limitations. The study was not designed to fully investigate the root causes of differences in rhGH management in children with CKD between institutions and individual nephrologists. There may have been a selection bias because physicians who were taking care of more children treated with rhGH (e.g., those attending CKD designated clinics, dialysis medical directors, transplant nephrologists) could have been more interested in study participation than those seeing fewer patients with advanced CKD and ESRD.

## Conclusions

This study found substantial variation in practice between pediatric nephrologists caring for short children with CKD. Practice size appears to be a major determinant of the logistic approach to rhGH management. Fear of injections was perceived as a most common obstacle to rhGH therapy initiation. Pediatric nephrologists believe that rhGH improves QOL in children with growth failure and CKD. Our data suggests that opportunities are available to standardize care to improve growth outcomes in children with CKD.

## Additional files

Additional file 1: Item S1. Midwest Pediatric Nephrology Consortium (MWPNC) collaborative research survey “Practice patterns of the recombinant human growth hormone use for the treatment of short stature in children with chronic kidney disease in North America”. original survey approved by the MWPNC Protocol Review Committee. (PDF 1021 kb)

Additional file 2: Figure S1. Side effects of growth hormone observed by the participating pediatric nephrologists within the last 5 years. Total number of participants *n* = 73. Figure S1 shows the number of reported side effects of recombinant human growth hormone therapy observed by the participating pediatric nephrologists within the past 5 years. The most commonly reported side effects were headache/benign intracranial hypertension and local reactions. (PNG 15 kb)

## Abbreviations

CKD: Chronic kidney disease
ESRD: End-stage renal disease
IGF-1: Insulin-like growth factor 1
IGFBP: Insulin-like growth factor binding protein
IQR: Interquartile range
KDOQI: Kidney Disease Outcomes Quality Initiative
MWPNC: Midwest Pediatric Nephrology Consortium
NAPRTCS: North American Pediatric Renal Trials and Collaborative Studies
QOL: Quality of life
rhGH: Recombinant human growth hormone
SDS: Standard deviation score

## References

[1] Rees L (2016). Growth hormone therapy in children with CKD after more than two decades of practice. Pediatr Nephrol.

[2] Seikaly MG, Salhab N, Gipson D, Yiu V, Stablein D (2006). Stature in children with chronic kidney disease: analysis of NAPRTCS database. Pediatr Nephrol.

[3] Rodig NM, McDermott KC, Schneider MF, Hotchkiss HM, Yadin O, Seikaly MG, et al. Growth in children with chronic kidney disease: a report from the Chronic Kidney Disease in Children Study. Pediatr Nephrol. 2014; 10.1007/s00467-014-2812-9 PMC4470271 24728472

[4] Harambat J, Bonthuis M, van Stralen KJ, Ariceta G, Battelino N, Bjerre A (2014). Adult height in patients with advanced CKD requiring renal replacement therapy during childhood. Clin J Am Soc Nephrol.

[5] Gerson AC, Wentz A, Abraham AG, Mendley SR, Hooper SR, Butler RW (2010). Health-related quality of life of children with mild to moderate chronic kidney disease. Pediatrics.

[6] Mekahli D, Ledermann S, Gullett A, Rees L (2014). Evaluation of quality of life by young adult survivors of severe chronic kidney disease in infancy. Pediatr Nephrol.

[7] Al-Uzri A, Matheson M, Gipson DS, Mendley SR, Hooper SR, Yadin O (2013). The impact of short stature on health-related quality of life in children with chronic kidney disease. J Pediatr.

[8] Fine RN, Yadin O, Moulton L, Nelson PA, Boechat MI, Lippe BM (1994). Five years experience with recombinant human growth hormone treatment of children with chronic renal failure. J Pediatr Endocrinol.

[9] Mahan JD, Warady BA (2006). Assessment and treatment of short stature in pediatric patients with chronic kidney disease: a consensus statement. Pediatr Nephrol.

[10] Koch VH, Lippe BM, Nelson PA, Boechat MI, Sherman BM, Fine RN (1989). Accelerated growth after recombinant human growth hormone treatment of children with chronic renal failure. J Pediatr.

[11] Seikaly MG, Salhab N, Warady BA, Stablein D (2007). Use of rhGH in children with chronic kidney disease: lessons from NAPRTCS. Pediatr Nephrol.

[12] van Huis M, Bonthuis M, Sahpazova E, Mencarelli F, Spasojevic B, Reusz G (2016). Considerable variations in growth hormone policy and prescription in paediatric end-stage renal disease across European countries-a report from the ESPN/ERA-EDTA registry. Nephrol Dial Transplant.

[13] Greenbaum LA, Hidalgo G, Chand D, Chiang M, Dell K, Kump T (2008). Obstacles to the prescribing of growth hormone in children with chronic kidney disease. Pediatr Nephrol.

[14] KDOQI Work Group. KDOQI Clinical Practice Guideline for Nutrition in Children with CKD: 2008 update. Executive summary. Am J Kidney Dis. 2009;53(3 Suppl 2):S11–104. 10.1053/j.ajkd.2008.11.017 19231749

[15] Rees L, Jones H (2013). Nutritional management and growth in children with chronic kidney disease. Pediatr Nephrol.

[16] Geisler A, Lass N, Reinsch N, Uysal Y, Singer V, Ravens-Sieberer U (2012). Quality of life in children and adolescents with growth hormone deficiency: association with growth hormone treatment. Horm Res Paediatr.

[17] Stephen MD, Varni JW, Limbers CA, Yafi M, Heptulla RA, Renukuntla VS (2011). Health-related quality of life and cognitive functioning in pediatric short stature: comparison of growth-hormone-naive, growth-hormone-treated, and healthy samples. Eur J Pediatr.

