# Supplementary material for: Approach to growth hormone therapy in children with chronic kidney disease varies across North America: the Midwest Pediatric Nephrology Consortium report
Source: BMC Nephrol. 2017 May 30;18:181. doi: 10.1186/s12882-017-0599-1 (PMC5450116; doi:10.1186/s12882-017-0599-1)
Supplement: Supplementary file 1 — Midwest Pediatric Nephrology Consortium (MWPNC) collaborative research survey “Practice patterns of the recombinant human growth hormone use for the treatment of short stature in children with chronic kidney disease in North America”. original survey approved by the MWPNC Protocol Review Committee. (PDF 1021 kb) [file 12882_2017_599_MOESM1_ESM.pdf]

MWPNC
MIDWEST PEDIATRIC NEPHROLOGY CONSORTIUM

COLLABORATIVE RESEARCH SURVEY

1. What is your primary affiliation?____

(for tracking purposes only, will not be disclosed)

2. How many full years have you been in practice after fellowship?__________

3. How many pediatric nephrologists are in your group (including yourself):________

4. How many of ***your*** patients are currently receiving growth hormone?____________(either enter the exact number or select one of the following categories. Do not include patients followed by your partners. For shared practices average) □ 0 □ 1-5 □ >5

5. Approximately, how many children are currently receiving chronic dialysis at your Center?__________________

(enter 0 if your Center does not provide dialysis services)

6. Approximately, how many kidney transplants are performed at your Center annually?__________________

(enter 0 if your Center does not perform transplants)

7. What is the role of endocrinology in growth hormone management in children with CKD at your Center?

□ In charge of most aspects of growth hormone therapy
□ Provides initial consultation for all children with CKD at the time of growth hormone therapy initiation, but subsequent management is primarily by nephrology.
□ Provides consultation for some challenging cases
□ Rarely involved in growth hormone therapy in children with CKD – it's managed primarily by nephrology
□ other (please specify):____

8. What is the nutritional support for children with pre-dialysis CKD and growth delay at your Center?

□ followed by a dedicated renal dietitian
□ followed by a pediatric dietitian
□ Most/all are only followed by a pediatric nephrologist. A limited number of patients are referred to dietitians.

9. What are the most common reasons that your short patients with CKD are not receiving growth hormone? (please check all that apply)

□ high likelihood of upcoming transplantation
□ medical contraindications
□ parents / provider perceives that risks outweigh benefit
□ family refusal [if so, circle the reason for the refusal: cost (insurance co-pays / deductible, out of pocket), concern about side effects, fear of injections, other:____]
□ difficulties with insurance approval
□ non-adherence
□ not applicable (I do not follow CKD patients who are short)
□ other (please specify):____

10. Who is typically obtaining prior authorization for insurance coverage of growth hormone therapy at your Center?

□ Attending physician
□ Fellow
□ Nurse practitioner
□ Physician assistant
□ Nurse
□ Social worker
□ Secretary / Clerk
□ other:____________________

11. The standard workup for growth hormone therapy initiation at your Center includes (please check all that apply):

□ bone age
□ hip and / or knee X-rays
□ ophthalmologic evaluation
□ thyroid studies
□ serum IGF-1
□ serum IGFBP3
□ endocrinology consult
□ other, please specify:___________________________

12. In your opinion, what are the medical benefits of growth hormone therapy in children with CKD besides its effect on linear growth (please check all that apply)?

□ improves quality of life
□ improves nutrition and appetite of children with CKD
□ increases lean body mass
□ improves physical function and decreases fatigue
□ improves bone mineral density
□ decreases risk of fractures
□ has beneficial anti-inflammatory activity
□ improves lipid profile
□ prevents / improves vascular disease in CKD
□ slows progression of CKD
□ other (please specify):__________________________

13. What side effects did you observe in your patients receiving growth hormone within the last 5 years? (Check all that applies)

□ Headache
□ Muscle pain
□ Arthralgia
□ Slipped capital femoral epiphysis
□ Avascular necrosis
□ Aggravation of secondary hyperparathyroidism
□ Faster than expected GFR decline
□ Hyperglycemia / insulin resistance / glucose intolerance
□ Injection site reaction
□ Benign intracranial hypertension / pseudo-tumor cerebri
□ Pancreatitis
□ Malignancy
□ Fluid retention
□ Allograft rejection (for transplant patients receiving growth hormone)
□ Fatigue / decreased school performance
□ Other:____
□ I did not treat any patients with growth hormone within the past 5 years
