# Supplementary figures and images for: Approach to growth hormone therapy in children with chronic kidney disease varies across North America: the Midwest Pediatric Nephrology Consortium report
Source: BMC Nephrol. 2017 May 30;18:181. doi: 10.1186/s12882-017-0599-1 (PMC5450116; doi:10.1186/s12882-017-0599-1)

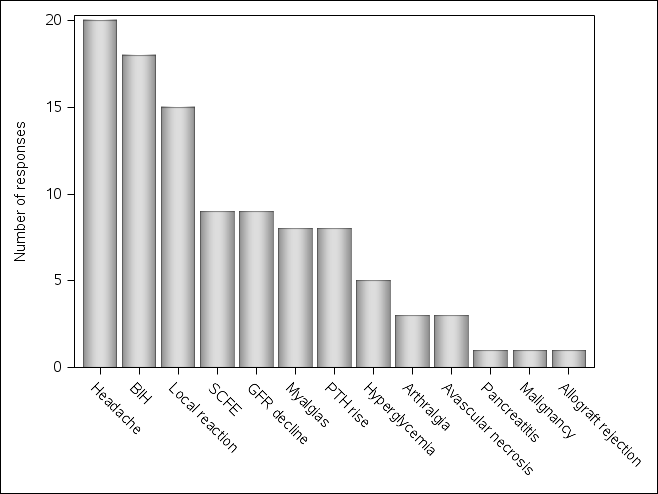

Supplement: Supplementary file 2 — Side effects of growth hormone observed by the participating pediatric nephrologists within the last 5 years. Total number of participants n = 73. Figure S1 shows the number of reported side effects of recombinant human growth hormone therapy observed by the participating pediatric nephrologists within the past 5 years. The most commonly reported side effects were headache/benign intracranial hypertension and local reactions. (PNG 15 kb) [file 12882_2017_599_MOESM2_ESM.png]
